# Comparison of three *Mycoplasma bovis* antibody ELISAs on serum from 3-5-week-old dairy calves using Bayesian latent class analysis

**DOI:** 10.1186/s13620-026-00354-2

**Published:** 2026-07-14

**Authors:** Jolien Vandewalle, Justine Clinquart, Wouter van Mol, Stan Jourquin, Bart Pardon

**Affiliations:** 1 Department of Internal Medicine, Reproduction and Population Medicine, Faculty of Veterinary Medicine, UGent Calf Health, Ghent University, Salisburylaan 133, Merelbeke, 9820 Belgium; 2 Animal Health Services Flanders (DGZ Vlaanderen), Industrielaan 29, Torhout, 8820 Belgium

**Keywords:** Purchase, *Mycoplasma bovis*, Diagnostics, Antibodies, Bayesian latent class analysis

## Abstract

**Background:**

Controlling *Mycoplasma bovis* is challenging in veal and dairy beef systems that source young calves from multiple origins. Pre‑purchase testing may help reduce pathogen introduction, with serology being the most accessible option. Additionally, given current EFSA recommendations to increase the minimum transport age to five weeks, this period represents the relevant time frame for pre‑purchase examination. However, antibody test performance in 3–5‑week‑old calves during acute infection is unknown. This study aimed to (1) compare the diagnostic accuracy of three commercial antibody ELISAs (ID‑screen, Bio K302 and Bio K432) in Belgian male dairy calves aged 3–5 weeks using Bayesian latent class models at manufacturer and optimized cutoffs, and (2) assess the effect of seroprevalence on predictive values.

**Results:**

A prospective diagnostic test accuracy study was conducted using 194 serum samples from calves aged 20–38 days. First, samples were analyzed with each ELISA according to manufacturer’s instructions. Next, test performance, as well as predictive values were evaluated using both manufacturer and optimized cutoff values. Using manufacturer’s cutoffs, sensitivities for ID‑Screen, Bio K302, and Bio K432 were 69.1%, 24.6%, and 68.7%, respectively, and specificities were 97.8%, 95.6%, and 97.9%. Optimized cutoffs improved sensitivities to 82.2%, 46.2%, and 88.9%, with specificities of 97.5%, 90.7%, and 89.3%. At a seroprevalence of 7.5%, positive predictive values (PPV) were 72.6% for Bio K432, 71.8% for ID‑Screen, and 31.2% for Bio K302, while negative predictive values (NPV) were similar for Bio K432 and ID‑Screen (both 97.5%) and slightly lower for Bio K302 (94%). At 20.8% seroprevalence, PPV increased for ID‑Screen (89.6%) compared with Bio K432 (68.6%) and Bio K302 (56.6%), while NPV remained high for ID‑Screen and Bio K432 (95.4% and 96.8%) but declined for Bio K302 (86.5%).

**Conclusions:**

During acute infection in 3–5‑week‑old calves, serological antibody testing against *M. bovis* shows strong potential as a rule‑in diagnostic tool. ID‑Screen and Bio K432 performed best, making them suitable for implementing feedback mechanisms to herds of origin, but they do not provide the strong rule-out capacity needed for effective screening.

## Introduction

*Mycoplasma bovis* is a major pathogen in cattle industries worldwide, leading to significant economic losses, animal welfare issues and high levels of antimicrobial use [1, 2]. Especially in the challenging veal and dairy beef production systems, the intensive purchase and commingling of calves from multiple origins lead to an almost unavoidable and systematic introduction of *M. bovis* into each production cycle [3]. In the context of sustainable livestock production, where improving animal welfare and reducing antimicrobial use have become key priorities, pre-purchase testing for *M. bovis* may help limit pathogen entry into veal and dairy beef systems. To achieve this, serological testing for antibodies against *M. bovis* represents the most accessible diagnostic option. Different antibody ELISA-kits are commercially available, including the commonly used ID-screen test (ID-Vet, Grabels, France) and three Bio-X tests, namely Bio K260, Bio K302 and Bio K432 (Bio-X Diagnostics, Rochefort, Belgium). In a recent study comparing the ID-screen, Bio K432 and Bio K302 kits using Bayesian latent class modeling, the ID-screen was shown to have both the highest sensitivity (Se) and specificity (Sp) when using optimized cutoffs over manufacturer’s cutoffs [4]. However, this study was based on serum collected from both calves and youngstock aged between one week and one year. This is important to consider because, as these assays can differ in their antigenic targets and immunoglobulin subtypes, their performance can be influenced by the calf’s age and immune status (e.g. colostral antibodies). In addition, given current European union recommendations to increase the minimum age for calf transport to five weeks, this age represents the relevant time frame for pre-purchase examination of calves [5]. Currently, different studies in the Belgian veal industry showed antibodies to be present upon arrival in 2–10% of the calves using the Bio K302 ELISA, with 15–20% of the calves already having pneumonia diagnosed with lung ultrasound at that time [6–8]. Meanwhile, a previous study in Belgium has shown that prevalence for *M. bovis* in dairy farms, based on bulk tank milk (BTM) is around 30% at herd-level [9]. Therefore, with the concern that the increased transportation age may result in a higher proportion of calves arriving with pneumonia and potentially a higher prevalence of *M. bovis* upon arrival, a reliable method to detect infected animals is warranted. However, there is currently no information available on performance of antibody tests in calves aged between three and five weeks, in the acute stage of the infection. Therefore, the objectives of this study were (1) to compare diagnostic accuracy of three commercial antibody ELISAs (ID-screen, Bio K302 and Bio K432) using serum samples from Belgian dairy male calves of 3–5 weeks old within a Bayesian latent class modeling framework, in which the unobserved latent classes represented animals with and without antibodies against *M. bovis*, both at manufacturer and optimized cutoff values and (2) to determine the effect of seroprevalence on predictive values.

## Materials and methods

### Study population and sampling strategy

A diagnostic test accuracy study was conducted on serum samples from male Holstein Friesian calves aged between 20 and 38 days, collected one week after arrival at the veal farm. To detect a 20% difference in Se and Sp with 80% power at an estimated seroprevalence of 30%, the minimum required sample size for a screening study was calculated to be 103 [10]. A total of 194 blood samples were collected from all calves present on a single veal calf farm, located in Geel, Antwerp (Belgium), in February 2025. The calves were surplus calves sourced from multiple conventional dairy herds before arrival at the veal farm. Blood samples were taken from the jugular vein of calves using a 21G needle and vacutainer (Venoject, Terumo, Leuven, Belgium). Samples were kept in a container (temperature of 4–7 °C) and arrived at the laboratory within 24 h after collection. Sampling was conducted during the peak incidence of bovine respiratory disease. The study protocol was approved by the Ethical Committee of the Faculty of Veterinary Medicine and Bioengineering from Ghent University under license number EC 2024-072. The study was conducted and reported following STARD-BLCM guidelines.

### Antibody ELISA tests and interpretation

All serum samples were analyzed with three indirect *M. bovis* ELISA kits: Bio K302 (Bio-X Diagnostics, Rochefort, Belgium), Bio K432 (Bio-X Diagnostics, Rochefort, Belgium) and ID-screen (ID-Vet, Grabels, France) following manufacturer’s instructions, as these are commercially available tests in the laboratories of the study region.

The conjugates used were protein G peroxidase, monoclonal α-IgG2 peroxidase and protein G peroxidase, respectively. The Sample-to-positive percentage (S/P%) for each sample and test was calculated using the following formula:

$$\:S/P\%\:=\left(\frac{ODsample-ODmean\:negative\:control}{ODmean\:positive\:control-ODmean\:negative\:control}\right)\times100$$

First, serum samples were labelled seropositive using manufacturer’s cutoffs. According to manufacturer’s cutoffs for Bio K302, Bio K432 and ID-screen, sera with an S/P% greater than or equal to 37%, greater than or equal to 30% or greater than or equal to 60% were considered seropositive, respectively. However, as described in the leaflet, ID-screen results can be semi-quantified and categorized as ‘+’ (S/P% ≥ 60), ‘++’ (≥ 80), ‘+++’ (≥ 110), and ‘++++’ (≥ 140). Therefore, the ID-screen results were secondly classified using these cutoff values. Finally, given that the Se of especially Bio X tests and the ID-screen at manufacturer defined cutoffs is often low, the results of the Bio K302, Bio K432 and ID-screen were further categorized using additional, experimentally defined cutoff values with decreasing intervals of 7–10% (range 60%-10%). This approach was used to explore whether lowering the S/P% cutoff values could improve diagnostic accuracy by increasing Se and thus reducing the number of false negatives.

### Bayesian latent class models – model development

In the absence of a gold standard for *M. bovis* antibody testing, a Bayesian latent class model (BLCM), in which the latent class represented the presence or absence of antibodies against *M. bovis*, was used to estimate diagnostic accuracy (Se and Sp) of the three tested antibody ELISAs applied on serum. A 3-test, 1-population model allowing for conditional dependence between ELISA tests was developed, based on the model described by Dendukuri and Joseph [11]. Dependency between ID-screen and Bio K302 was considered due to their use of the same conjugate, protein G peroxidase, in contrast to Bio K432, which uses a monoclonal α-IgG2 peroxidase.

### Bayesian latent class models – prior distribution determination

In BLCM, incorporating informative priors into the model is a way of providing the model with previously described information about disease prevalence and the diagnostic tests’ Se and Sp to reduce parameter uncertainty. Four models were run for conditional dependence. In the first model, all prior information was set at uninformative: β (1, 1). The priors were defined by beta distributions calculated with the “EpiR” package [12]. In the second model the Se (β (5.608, 1.113)) and Sp (β (10.522, 3.562)) of the ID-screen were added. In the third model, Se (β (5.739, 10.365)) and Sp (β (5.425, 1.040)) of the Bio K302 were added and in the fourth model, Se (β (6.949, 1.698)) and Sp (β (7.17, 1.802)) of the Bio K432 were added. Information on Se and Sp of the Bio K302, Bio K432 and ID-screen were obtained from Bokma et al. [4].

### Bayesian latent class models – model analysis

All analyses were conducted in R (version 4.3.1) using RStudio (R Core Team, 2021). First, to determine the diagnostic accuracy of the manufacturer’s cutoffs, a dependent model as reported by Dendukuri and Joseph [11] was executed by “rjags” [13] whereby ID-screen and Bio K302 were considered conditionally dependent and Bio K432 independent. To account for conditional dependence between Bio K302 and ID-screen, covariance parameters were estimated for antibody-positive (covp) and antibody-negative (covn) animals. The model fit was evaluated by using the deviance information criterion (DIC). When the difference in DIC between models is less than two, models are considered not to be statistically different [14]. A modified code from Cheung et al. [15] was used consisting of 3 chains for which iterations were specified. Diagnostic assessments, including autocorrelation and trace plots, were generated using the “mcmc plots” package, and the effective sample size of each parameter was estimated with the “Coda” package [16]. Because autocorrelation was frequently observed, a thinning factor of 10 was applied. The total number of retained iterations (200,000) was determined to ensure a minimum effective sample size of 10,000. A burn-in period of 10,000 iterations was used. The posterior median and 2.5–97.5% credible intervals (CIs) were obtained for Se, Sp and prevalence. To ensure the robustness for models incorporating specific priors, a Se analysis was performed by shifting the mode by 15%, as recommended by Johnson et al. [17]. The posterior estimates from these alternative models were verified to have substantial overlap with the 95% credible interval of the main model. Additionally, the posterior probability that the Se of test A exceeded that of test B was computed. Secondly, four dependent models without prior information were run for ID-screen S/P% cutoff 60%, 80%, 110% and 140% compared to Bio K302 and Bio K432 to determine the optimal manufacturer cutoff value for ID-screen. Third, to determine optimal cutoff values for all three ELISA kits, dependent models without prior information were run for a range of alternative S/P% cutoff values for Bio K302 (30%-10%), Bio K432 (20%-10%) and ID-screen (50%-10%). The optimal cutoff value for each diagnostic test was determined based on the results of the BLCM and the highest Youden’s index (sensitivity + specificity – 1). Finally, a dependent model incorporating the optimal S/P% cutoff value for each test was run. Additionally, the positive likelihood ratio (LR+), negative likelihood ratio (LR-), positive predictive value (PPV), and negative predictive value (NPV) were determined once for the manufacturer’s cutoffs and once for the optimized cutoff values. For the calculations of LR + and LR-, the following formulas were used: $\:\frac{sensitivity}{1-specificity}$ and $\:\frac{1-sensitivity}{specificity}$, respectively. PPV and NPV were determined using Winepi [18]. Finally, plots of PPV and NPV were generated in R (version 4.3.1).

## Results

### Seroprevalence

In total, 5.2% (10/194) of the serum samples were positive for *M. bovis* antibodies using Bio K302_≥ 37_, while this was 6.2% (12/194) and 6.7% (13/194) for Bio K432_≥ 30_ and ID-screen_≥ 60_, respectively. Table 1 shows the proportion of seropositive samples when categorizing results of the ID-screen, Bio K302 and Bio K432 using different cutoff values. Among samples positive by Bio K302, 20.0% (2/10) were also positive by ID‑screen and 20% (2/10) were also positive by Bio K432. Conversely, among samples positive by ID‑screen, 46.2% (6/13) were also positive by Bio K432.

**Table 1:** Overview of the number of seropositive samples from calves for three Mycoplasma bovis antibody ELISA tests (ID-screen, Bio K302, Bio K432), using different cutoff values for the sample-to-positive percentage (S/P%)

Test	S/*P*% cutoff	Seropositives (%)
Bio K302	≥ 37%^*^	5.2 (10/194)
	≥ 30%	7.2 (14/194)
	≥ 20%	16.5 (32/194)
	≥ 10%	36 (70/194)
Bio K432	≥ 30%^*^	6.2 (12/194)
	≥ 20%	9.8 (19/194)
	≥ 10%	26.3 (51/194)
ID-screen	≥ 140%^**^	0.5 (1/194)
	≥ 110%^**^	1.6 (3/194)
	≥ 80%^**^	4.6 (9/194)
	≥ 60%^*^	6.2 (13/194)
	≥ 50%	10.8 (21/194)
	≥ 40%	14.4 (28/194)
	≥ 30%	18.6 (36/194)
	≥ 20%	27.8 (54/194)
	≥ 10%	40.7 (79/194)

*Manufacturer’s cutoffs: Bio K302_≥ 37_, Bio K432_≥ 30_, ID-screen_≥ 60_. **ID-screen results can also be semi-quantified and categorized as ‘+’ (S/P% ≥ 60), ‘++’ (≥ 80), ‘+++’ (≥ 110), and ‘++++’ (≥ 140). Therefore, the ID-screen results were secondly classified using these cutoff values. Additional cutoffs were applied in 7–10% decreasing intervals (60%-10%) to assess whether lowering the S/P% threshold could improve diagnostic Se

### Bayesian latent class modelling

First, the ID-screen, Bio K432 and Bio K302 tests were compared using cutoff values provided by the manufacturer. Four models were run with conditional dependence, each with different priors.

Conditional dependence was modeled between Bio K302 and ID-screen. The covariance among antibody-negative animals (covn) was positive (Median: 0.9%, 95% credible interval (CI): 0.02%-2.9%), indicating residual positive dependence between the assays. In contrast, the covariance among antibody-positive animals (covp) was negative (-8.6%, 95%CI: -20.2%-0.8%), although the credible interval included zero, suggesting limited evidence for meaningful residual dependence. All these models showed similarities for both Se and Sp (Table 2). Based on DIC, model three achieved the lowest DIC (33.8), followed by model one with a DIC of 34.4. Given the small difference in DIC (0.6) between model one and three, and model one did not include any prior information, model one was used for further Bayesian latent class modelling (Table 2, bold). Model one showed a moderate Se for ID-screen (69.1%, 95% credible interval (CI): 37.1%-94.5%) and Bio K432 (68.7%, 95%CI: 35.9%-97.8%) and low Se for Bio K302 (24.6%, 95%CI: 5.8%-53.3%). The Sp was high for ID-screen (97.8%, 95%CI: 93.9%-99.8%), Bio K432 (97.9%, 95% CI: 94.5%-99.8%) and Bio K302 (95.6%, 95%CI: 91.5%-98.8%). Although the confidence intervals overlapped across all models, the ID-screen showed a numerically higher performance estimate, similar to the Bio K432, while both performed better than the Bio K302. This was supported by posterior probabilities indicating an approximately 97% probability that Bio K432 had a higher Se than Bio K302, and about a 96% probability that ID-screen had a higher Se than Bio K302.

**Table 2:** Posterior medians and 95% credible intervals [CI95] for four conditional dependent models for the sensitivity (Se) and specificity (Sp) of three Mycoplasma bovis antibody ELISA tests (Bio K302, Bio K432 and ID-screen) on serum from calves while using manufacturer cutoff values for the sample-to-positive percentage (S/P%)

Test	Parameter	Model 1^a^	Model 2^b^	Model 3^c^	Model 4^d^
Conditional dependent					
Bio K302_≥ 37_	Se (%) [CI95]	**24.6 [5.8–53.3]**	19.5 [3.1–46.8]	29.7 [14.5–48.4]	23.4 [4.7–52.4]
Bio K302_≥ 37_	Sp (%) [CI95]	**95.6 [91.5–98.8]**	94.7 [90.5–97.8]	95.9 [92.0-98.9]	95.3 [91.3–98.2]
Bio K432_≥ 30_	Se (%) [CI95]	**68.7 [35.9–97.8]**	79.5 [43.5–99.0]	67.3 [35.3–97.5]	80.2 [52.7–97.4]
Bio K432_≥ 30_	Sp (%) [CI95]	**97.9 [94.5–99.8]**	97.5 [94.1–99.6]	97.9 [94.6–99.8]	97.3 [93.9–99.4]
ID-screen_≥ 60_	Se (%) (CI95]	**69.1 [37.1–94.5]**	80.3 [52.9–97.3]	66.4 [36.9–91.5]	73.1 [40.9–95.9]
ID-screen_≥ 60_	Sp (%) [CI95]	**97.8 [93.9–99.8]**	95.9 [92.1–98.5]	97.9 [94.0-99.8]	97.3 [93.6–99.6]
	Prev (%) [CI95]	**7.5 [3.3–14.3]**	6.0 [2.6–11.3]	7.7 [3.4–14.7]	6.5 [2.9–12.1]
	Covn* (%)	**0.9 [0.02–2.9]**	1.4 [0.15–3.8]	0.8 [0.02–2.8]	0.9 [0.06-3.0]
	Covp** (%)	**-8.6 [-20.2-0.8]**	-7.3 [-18.8-0.3]	-10.2 [-20.6-0.8]	-8.7 [-20.5-0.3]
	DIC	**34.4**	38.1	33.8	34.6

^a^Model 1: no informative priors (β (1,1))

^b^Model 2: informative prior on ID-screen sensitivity (β (5.608, 1.113); mode 97.6%; 5th percentile 50%) and specificity (β (10.522, 3.562); mode 78.8%; 5th percentile 50%)

^c^Model 3: informative prior on Bio K302 sensitivity (β (5.739, 10.365); mode 33.6%; 5th percentile 15%) and specificity (β (5.425, 1.040); mode 99.1%; 5th percentile 50%)

^d^Model 4: informative prior on Bio K432 sensitivity (β (6.949, 1.698); mode 89.5%; 5th percentile 50%) and specificity (β (7.17, 1.802); mode 88.5%; 5th percentile 50%)

*Covn: covariance among antibody-negative animals

**Covp: covariance among antibody-positive animals

Note: The first model with values in bold was used for further Bayesian latend class modelling

The Se analysis confirmed the model’s robustness to prior specification, as the posterior estimates from the alternative model (by shifting the mode by 15%) fell within the 95% credible interval of the main model. Secondly, four dependent models without prior information were run for ID-screen S/P% cutoff 60%, 80%, 110% and 140% compared to Bio K302 and Bio K432. When increasing the cutoff to 80%, this resulted in a really small increase in Sp (98.2%, 95%CI: 95.1%-99.8%), but a decrease in Se (61.8%, 95%CI: 26.1%-94.4%). However, for cutoff 110% and 140%, only 3 and 1 positive test results were obtained, which did not allow the models to be executed. Third, dependent models without prior information were run for a range of alternative S/P% cutoff values to increase the low Se of Bio K302 (24.6%) and moderate Se of Bio K432 (68.7%) and ID-Screen (69.1%). The results of these models are shown in Table 3. The highest Youden’s index (J = 0.560) was obtained for Bio K302 with a cutoff value of 20%, with a Se of 67.9% (39.2%-88.1%) and a Sp of 88.2% (82.1%-95.0%). For Bio K432, the highest Youden’s index (J = 0.729) was reached when the cutoff was set at 10%, with a Se of 90.4% (70.0%-99.5%) and a Sp of 82.4% (74.8%-95.9%), whereas for the ID-screen the maximum Youden’s index was reached at a 30% cutoff (J = 0.803), resulting in a Se of 89.0% (69.2%-98.5%) and a Sp of 91.3% (83.5%-99.1%), respectively. Finally, a dependent model incorporating the optimal S/P% cutoff value for each test was run (Table 4). This model, including ID-screen_≥ 30_, Bio K302_≥ 20_ and Bio K432_≥ 10_, showed a moderate Se for ID-screen (82.2%, 95%CI: 54.7%-98.5%) and Bio K432 (88.9%, 95%CI: 72.0%-99.3%), but rather low Se for Bio K302 (46.2%, 95%CI: 29.2%-63.8%). The Sp was high for ID-screen (97.5%, 95%CI: 92.7%-99.9%), Bio K432 (89.3%, 95%CI: 82.1%-98.6%) and Bio K302 (90.7%, 95%CI: 84.8%-95.4%). The sensitivity analysis demonstrated that estimates for Se, Sp and prevalence are mostly stable across models. Only for the Se estimate of Bio K302, a more pronounced deviation from the 95% CI of the initial model was observed.

**Table 3:** Posterior medians and 95% credible intervals [CI95] for a dependent test for the determination of sensitivity (Se) and specificity (Sp) of three Mycoplasma bovis antibody ELISA tests (Bio K302, Bio K432 and ID-screen) and seroprevalence (prev) on serum from calves while using optimized cutoff values for the sample-to-positive percentage (S/P%) of the Bio K302, ID-screen and Bio K432

Test	S/*P*% cutoff	Se (%) [CI95]	Sp (%) [CI95]	Prev (%) [CI95]	Youden’s index (J)
Bio K302	≥ 37%	24.6 [5.8–53.3]	95.6 [91.5–98.8]	7.5 [3.3–14.3]	0.201
	≥ 30%	39.3 [15.5–67.7]	94.9 [90.5–98.7]	8.0 [3.8–14.9]	0.341
	**≥ 20%**	**67.9 [39.2–88.1]**	**88.2 [82.1–95.0]**	**8.9 [4.4–17.4]**	**0.560**
	≥ 10%	86.2 [61.1–98.0]	69.0 [61.4–76.8]	9.3 [4.1–18.0]	0.550
Bio K432	≥ 30%	68.7 [35.9–97.8]	97.9 [94.5–99.8]	7.5 [3.3–14.3]	0.664
	≥ 20%	77.0 [46.2–98.3]	95.3 [90.8–99.3]	8.3 [3.9–16.0]	0.723
	**≥ 10%**	**90.4 [70.0-99.5]**	**82.4 [74.8–95.9]**	**12.6 [6.6–27.6]**	**0.729**
ID-screen	≥ 60%	69.1 [37.1–94.5]	97.8 [93.9–99.8]	7.5 [3.3–14.3]	0.665
	≥ 50%	72.4 [43.6–94.0]	94.9 [89.4–99.3]	9.3 [4.0-17.4]	0.673
	≥ 40%	86.0 [62.2–98.3]	94.1 [87.4–99.5]	11.3 [5.0-19.7]	0.799
	**≥ 30%**	**89.0 [69.2–98.5]**	**91.3 [83.5–99.1]**	**12.8 [5.5–22.7]**	**0.803**
	≥ 20%	92.2 [73.2–99.0]	81.7 [72.4–96.9]	12.7 [5.1–28.8]	0.738
	≥ 10%	96.6 [83.2–99.9]	78.3 [64.6–98.0]	25.4 [11.6–43.4]	0.741

Optimal cutoff values for Bio K302, Bio K432 and ID-screen are highlighted in bold

**Table 4:** Posterior medians and 95% credible intervals [CI95] for a dependent test for the determination of sensitivity (Se) and specificity (Sp) of three Mycoplasma bovis antibody ELISA tests (ID-screen, Bio K302 and Bio K432) and seroprevalence (prev) on serum from calves while using optimized cutoff values for the sample-to-positive percentage (S/P%) (final model)

Test	S/P% cutoff	Se (%) [CI95]	Sp (%) [CI95]	Prev (%) [CI95]	Youden’s index (J)
ID-screen	≥ 30%	82.2 [54.7–98.5]	97.5 [92.7–99.9]	20.8 [13.6–31.9]	0.794
Bio K302	≥ 20%	46.2 [29.2–63.8]	90.7 [84.8–95.4]	20.8 [13.6–31.9]	0.368
Bio K432	≥ 10%	88.9 [72.0-99.3]	89.3 [82.1–98.6]	20.8 [13.6–31.9]	0.782

**Fig. 1:**
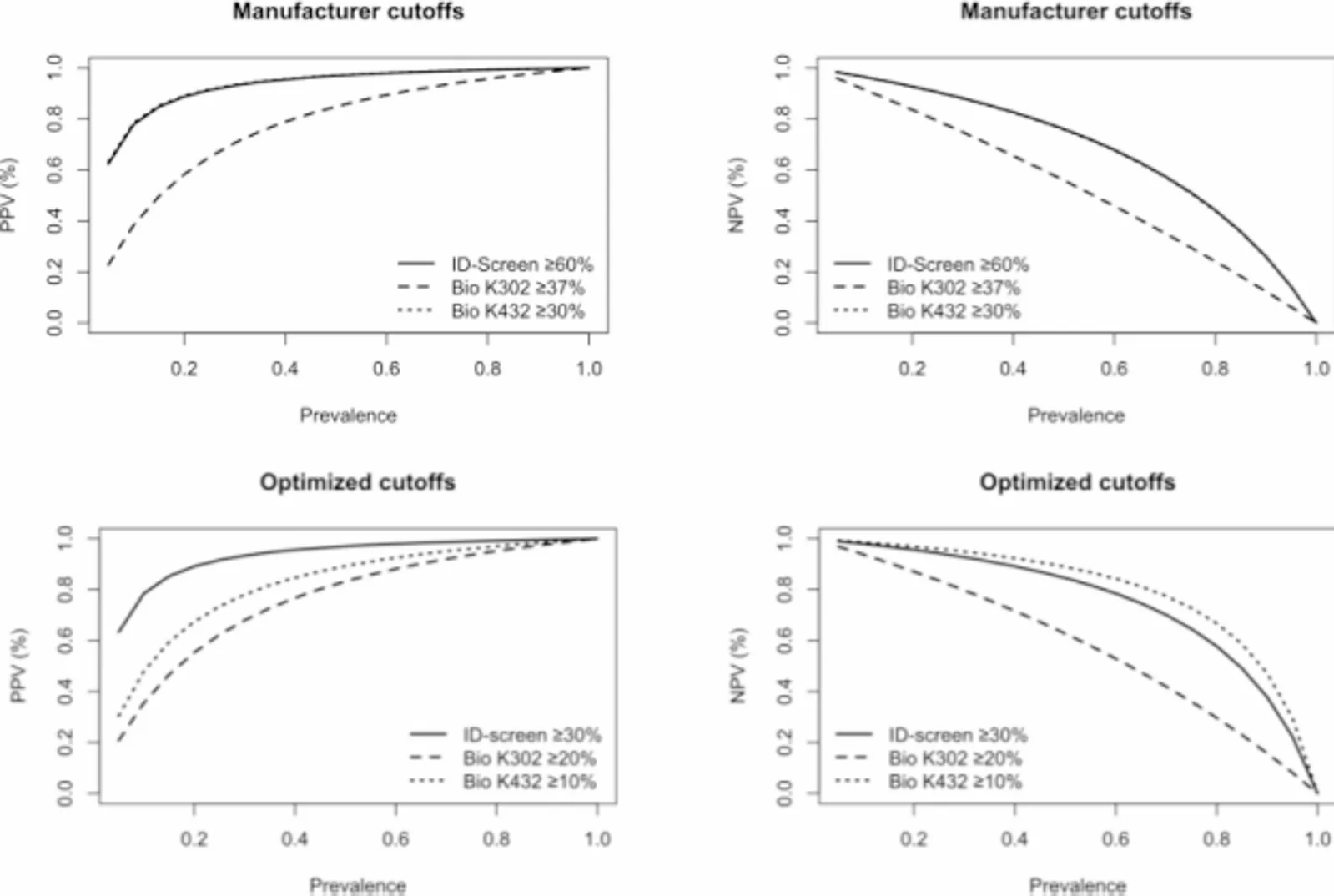
Positive (PPV) and negative predictive values (NPV) as a function of seroprevalence for three ELISA’s at manufacturer’s (top) and optimized (bottom) cutoffs. Manufacturer’s cutoffs used prevalence, Se and Sp from model 1. Optimized cutoffs used values from the final model

Additionally, likelihood ratios as well as predictive values were calculated for each ELISA, once for the manufacturer’s cutoff values (Model 1) and once for the optimized cutoff values (Final model) (Table 5). With manufacturer’s cutoffs, the LR + for Bio K432, ID-screen and Bio K302 were 32.7, 31.4 and 5.6, respectively, while the LR- were 0.32, 0.32 and 0.79, respectively. When optimized cutoffs were applied, the LR + for Bio K432, ID-screen and Bio K302 were 8.31, 32.9 and 4.97, respectively, while the LR- were 0.12, 0.18 and 0.59, respectively. In a low-prevalence population (7.5%), PPV values for Bio K432 and ID-screen were 72.6% and 71.8%, respectively, whereas the PPV value for Bio K302 was markedly lower (31.2%). In the same low-prevalence population, NPV values for Bio K432 and ID-screen were identical (97.5%), while NPV value for Bio K302 was 94%. When looking at a population with a prevalence of 20.8%, PPV values for Bio K432 and ID-screen were 68.6% and 89.6%, respectively, while PPV value for Bio K302 was 56.6%. NPV values for Bio K432 and ID-screen were 96.8% and 95.4% respectively, while NPV value for Bio K302 was 86.5%. The predictive value curves are shown in Fig. 1.

**Table 5:** Seroprevalence (prev), positive (LR+) and negative likelihood ratio’s (LR-) and positive (PPV) and negative predictive values (NPV) of three Mycoplasma bovis antibody ELISA tests (Bio K432, ID-screen and Bio K302) on serum from calves while using both manufacturer’s and optimized cutoff values for the sample-to-positive percentage (S/P%)

Test	Prev (%) (CI95)	LR+	LR-	PPV (%)	NPV (%)
Manufacturer cutoffs					
Bio K432_≥ 30_	7.5 [3.3–14.3]	32.7	0.32	72.6	97.5
ID-screen_≥ 60_	7.5 [3.3–14.3]	31.4	0.32	71.8	97.5
Bio K302_≥ 37_	7.5 [3.3–14.3]	5.6	0.79	31.2	94.0
Optimized cutoffs					
Bio K432_≥ 10_	20.8 [13.6–31.9]	8.31	0.12	68.6	96.8
ID-screen_≥ 30_	20.8 [13.6–31.9]	32.9	0.18	89.6	95.4
Bio K302_≥ 20_	20.8 [13.6–31.9]	4.97	0.59	56.6	86.5

## Discussion

Testing calves around the time of purchase, either before or shortly after arrival, can provide valuable information to improve control and prevention strategies for *M. bovis* in veal facilities. Although serum antibody testing is the most feasible in practice, there is currently no information on its performance in 3–5-week-old male calves, an age category of particular interest in regard of the new transport age allowed (EFSA, 2023) [5]. Also, test performance in terms of predictive value is influenced by seroprevalence, which naturally varies between populations. Therefore, this study aimed to evaluate the performance of three commonly used antibody ELISA tests in 3-5-week-old calves, using both manufacturer’s and optimized cutoff values, and to determine seroprevalence effects on predictive values. A gold standard for *M. bovis* antibody testing is lacking, therefore a BLCM analysis was used, in which the latent class represented the presence or absence of antibodies against *M. bovis*. The authors like to underscore that this study was designed for the detection of antibodies, whereas information of how the presence of antibodies is associated with infectious status and the possibility to spread the infection to other animals, as well as the carrier state is currently incompletely understood. Therefore, the results of this study should be interpreted as detection of antibodies against *M. bovis*, regardless of whether their presence signifies an infectious risk.

The main finding of this study is that Bio K432 and ID-screen performed almost identical, whereas Bio K302 showed clearly inferior Se, confirming lower diagnostic accuracy in the present dataset. When looking at the results of the dependent models for manufacturer’s S/P% cutoff values, model 1 served as the main reference because it relies on uninformative priors and is therefore less influenced by prior assumptions. This ensures that the results are driven as much as possible by the observed data rather than by external prior information. In Model 1, the ID-screen test showed the highest median Se (69.1%), closely followed by Bio K432 (68.7%), whereas Bio K302 had a substantially lower Se (24.6%). Although CI overlapped, indicating reduced evidence for a difference between the estimates, the posterior estimates suggest that a lower Se for Bio K302 is more plausible than equivalence. This is supported by posterior probabilities of approximately 97% for Bio K432 and 96% for ID-screen having higher Se than Bio K302. In comparison with the uninformative model (Model 1), the models incorporating informative priors (Models 2–4) showed a 5–10% increase in Se, but CIs remained wide, indicating reduced data support and strong prior influence. A likely explanation for this uncertainty is the low seroprevalence in the studied population, resulting in few positive cases. As a result, there were relatively few true-positives and false-negative results available to reliably estimate Se. This limited amount of information reduced the data’s contribution to the posterior distributions, making the influence of the prior distributions more apparent. In contrast, Sp estimates remained consistently high (approximately 94–98%) across all models, with overlapping CI, suggesting similar performance. The minimal change in Sp estimates after adding informative priors indicates that the data contained sufficient information to estimate Sp robustly. Overall, our findings are in contrast with a previous study on serum samples from youngstock where they reported Se and Sp results of 97.6% and 78.8% for ID-screen, respectively [4]. Disagreement in diagnostic accuracy between studies and tests is most likely due to differences in the examined population. Our study included 3-5-week-old calves in the acute stage of the infection, whereas Bokma et al. [4] dealt with one week up to one year old animals with unclear information on duration since time of infection. For *M. bovis*, seroconversion can be expected within a range of 9–21 days with peaks between 23 and 40 days, but in some cases can be delayed for up to four months [19, 20]. This could explain the low proportion of calves with detectable antibodies one week after arrival in our study. The small number of calves that was seropositive can be explained by two possible reasons: either they were infected very early at their farm of origin and have already developed antibodies themselves, or they had maternal antibodies. It’s important to consider that the nature of the antibody response may also be responsible for differences in diagnostic accuracy. The ELISA’s that were evaluated are using different conjugates (e.g. protein G peroxidase for ID-screen and Bio K302 and monoclonal α-IgG2 peroxidase for Bio K432, respectively), that can result in different test outcomes depending on the antibody response that was measured (e.g. total ruminant IgG and IgG2, respectively). During *M. bovis* infections, IgG1 production generally predominates over IgG2. However, IgG2 is also present and may differ from IgG1 in binding capacity and response dynamics [21, 22]. In calves, the detection of IgG1 could also possibly come from maternally derived antibodies via colostrum, whereas the detection of IgG2 is due to production by the calf itself in response to an *M. bovis* infection [22]. Therefore, the Bio K432 has potential to only detect the calf’s own humeral response to infection. Another factor that could contribute to disagreement in different diagnostic test accuracy is variation in test characteristics, which may influence susceptibility to certain *M. bovis* variants or cross-reactivity with other antigens [23]. The positive covariance observed among antibody-negative animals may reflect shared assay characteristics, such as non-specific ELISA reactivity or cross-reactive antibodies. Although the posterior median covariance among antibody-positive animals was negative, the associated uncertainty was substantial and the credible interval overlapped zero. Therefore, this finding should be interpreted cautiously and may reflect estimation uncertainty rather than a biologically meaningful inverse relationship between ID-screen and Bio K302. Nevertheless, it’s interesting to observe that Bio K432 and ID-screen showed similar performance, even though they both use a different conjugate. This suggests that also broader aspects of assay design (e.g. epitope presentation and optical density behavior) could play an important role in determining test performance.

In addition, we further assessed optimized cutoff values for each ELISA to determine the most suitable threshold for screening calves aged three to five weeks for *M. bovis*. Cutoff values were chosen to maximize the risk of falsely classifying infected animals as free from infection. Lowering cutoffs resulted in an increase in Se for all three tests; substantially for Bio K432 (88.9%) and ID-screen (82.2%), but only moderate for Bio K302 (46.2%). For Sp, a clearer distinction was observed between the ID-screen test (97.5%), Bio K432 (89.3%), and Bio K302 test (90.7%); however, there is still narrowly overlap between CI. The likelihood ratios further highlight these differences. At manufacturer’s cutoffs, Bio K432 and ID-screen showed high LR+ (32.7 and 31.4, respectively), providing strong rule-in evidence. In contrast, the Bio K302 showed a substantially lower LR+ (5.6), suggesting only moderate rule-in evidence. Regarding LR−, Bio K432 and ID-screen both showed values of 0.32, moderately reducing the probability of being seropositive when the test result is negative, whereas Bio K302 performed poorly (0.79). When optimized cutoffs were applied, especially LR− values improved (Bio K432 = 0.12; ID-screen = 0.18; Bio K302 = 0.59) indicating a stronger ability to rule-out seropositivity. However, Bio K302 remained comparatively weaker. Overall, these findings suggest that Bio K432 and ID-screen provide stronger diagnostic evidence for ruling in being seropositive through high LR+ values both for manufacturer’s and optimized cutoffs. In contrast, both at manufacturer’s and optimized cutoffs, the three tests only had weak (Bio K302) to moderate (ID-screen and Bio K432) suitability as a screening (rule-out) test in this study population. Based on the abovementioned likelihood ratios, both Bio K432 and ID-screen ELISA results could contribute to an *M. bovis* herd status classification (e.g. negative, suspect, positive). High LR+ from Bio K432 or ID-screen supports positive or suspect *M. bovis* herd status when ≥ 1 animals test positive, while improved LR − at optimized cutoffs strengthens negative herd certification by reducing false negatives. However, persistent moderate LR− values limit standalone reliability for official *M. bovis*-free herd declaration.

Finally, given that seroprevalence significantly affects the performance of the tests, it’s useful to examine how performance alters as a function of seroprevalence. In this context, seroprevalence refers to the proportion of animals with detectable antibodies against *M. bovis*, which was also the latent condition in our study. At herd level in dairy cattle, a previous study in Belgium has shown that disease prevalence (seroprevalence + antigen detection) is around 30% [7]. However, as it is currently unknown what the within herd prevalence in infected herds is and not all calves originate from infected herds, the seroprevalence in the current study was assumed to be unknown. To account for this uncertainty, and to illustrate differences between different cutoff values, the predictive value curves (Fig. 1) were calculated. These curves (5-100% prevalence) reveal clear differences between manufacturer’s and optimized cutoffs. At manufacturer’s cutoffs, ID-screen and Bio K432 showed nearly identical, high PPV curves, while Bio K302’s was markedly lower. Even a small absolute difference in Sp leads to a substantially higher false-positive burden at low prevalence, resulting in a visibly lower PPV. Optimized cutoffs further separate ID-screen above both Bio-X tests. This difference can be explained by ID-screen’s very high Sp (97.5%), which results in few false positives and therefore maintains a high PPV, even at low prevalence. Especially at a prevalence of 20.8%, even small losses in Sp of both Bio-X tests create more false positives, which depress PPV. At higher seroprevalence levels, the difference between the three tests decreases; however, this only occurs at unrealistically high seroprevalence levels above 80%. As NPV is largely driven by Se, the difference between ID-screen and Bio K432 on one hand and Bio K302 on the other hand, can be explained by this factor. NPV curves overlap for ID-screen and Bio K432 at manufacturer’s cutoffs (Bio K302 drops faster from low Se), with slower declines after optimization reflecting higher Se. However, NPV is also driven by prevalence and given the low seroprevalence (7.5%-20.8%) in our population, NPV remains consistently high across all three tests. However, the differences again become smaller with increasing prevalence, especially above 50%. In summary, optimized cutoffs mainly improve PPV for ID-screen at low prevalence while maintaining high NPV across all tests. Under the low seroprevalence conditions observed in this study, the NPV likely primarily reflects the low seroprevalence rather than strong negative discriminatory performance, as Se remained limited across all three assays. Therefore, extrapolation of the reported PPV and NPV to populations with differing seroprevalences should be considered with caution.

Finally, as this study was performed under field-conditions, the findings are subjected to a number of limitations. First, because the calves are aged between three and five weeks old, the detection of IgG1 could also possibly come from the colostrum of a cow previously exposed to *M. bovis*, which could lead to animals being wrongly identified as having antibodies after infection (false positive). However, it is still not known what the proportion of maternal antibodies is and whether maternal antibodies have a protective function. Also, because the calves originated from multiple source herds, their pre-existing exposure histories were likely heterogeneous. As the exact number of source herds was not recorded, this potential source of variation could not be accounted for in the analysis and should be considered when interpreting the serological findings. Another important limitation was that not all calves may already have developed antibodies at the time of sampling, which means some infected calves might have been missed. Therefore, it would be interesting to assess whether the presence of antibodies correlates with reduced, absent or ongoing shedding of the pathogen. A third limitation relates to uncertainties on the estimated prevalence of 30% used for sample size calculation. This estimate was based on BTM results rather than individual calf results, so it may overestimate the true seroprevalence in calves due to either not yet being infected or not yet having seroconverted [19, 20]. Seropositivity in veal calves upon arrival has been described as low as 2–10%, which is much lower than the a priori estimated prevalence in our study [6, 7]. However, since these studies used the Bio K302 kit (low Se) and calves were sampled earlier in life (lower seroconversion rate), the true seroprevalence during the acute phase of infection remains uncertain. Nevertheless, a lower true seroprevalence would mean that the a priori determined sample size may have been insufficient to detect a biologically relevant difference within this study population. With the estimated seroprevalence of around 10% and 20% for the manufacturer’s and optimized cutoffs, respectively, the available sample size only allowed for a detection of a 30% difference between tests, which is reflected by the wide and overlapping CI [10]. This particularly affects sensitivity estimation, as relatively few positive animals were available to inform the model. Despite this uncertainty, both ID-screen and Bio K432 consistently showed high Sp and mainly appear to have value as rule-in tests, and with their almost identical Sp, it is unlikely that simply increasing the study power would result in a different study outcome.

## Conclusion

In the studied population of 3-5-week-old calves in the acute stage of infection, ID-screen and Bio K432 performed similarly and substantially better than Bio K302. However, Se and LR- of all three tests remained too low to serve as a reliable pre-purchase screening test, even after adjusting cutoffs to improve Se. LR + was highest at manufacturer’s cutoffs, supporting rule-in potential, whereas predictive values were strongly dependent on seroprevalence, potentially limiting their reliability as standalone diagnostic measures under low seroprevalence conditions in this study. Therefore, antibody testing mainly demonstrates potential as a rule-in test for control and prevention, creating the possibility for a feedback mechanism towards the herds of origin. Although, they may have value in control programs, the industry would particularly benefit from tests with strong rule-out capacity, for which these antibody ELISAs do not appear sensitive enough.

## Data Availability

Data is available from the corresponding author on reasonable request.

## Abbreviations

EFSA: European Food Safety Authority
ELISA: Enzyme-Linked Immunosorbent Assay
M.: bovis Mycoplasma bovis
Se: Sensitivity
Sp: Specificity
BTM: Bulk tank milk
S/P%: Sample-to-positive percentage
BLCM: Bayesian latent class model
COVP: Covariance in antibody-positive animals
COVN: Covariance in antibody-negative animals
DIC: Deviance information criterion
CI: Credible interval
LR+: Positive likelihood ratio
LR-: Negative likelihood ratio
PPV: Positive predictive value
NPV: Negative predictive value
95%: CI 95% credible interval
